# Anterior Chamber Angle Evaluation with Fourier-Domain Optical Coherence Tomography

**DOI:** 10.1155/2012/103704

**Published:** 2012-07-02

**Authors:** Matthew Bald, Yan Li, David Huang

**Affiliations:** Center for Ophthalmic Optics & Lasers, Casey Eye Institute and Department of Ophthalmology, Oregon Health & Science University, Portland, OR 97239-4197, USA

## Abstract

New advances in anterior segment optical coherence tomography (OCT) technology development allow visualizing the anterior chamber angle of the eye with high speed and high resolution. Fourier-domain (FD) OCT instruments working at 840 nm can reliably identify fine angle structures such as the Schwalbe's line. This paper demonstrates quantitative angle assessment with 840 nm FD-OCT and provides diagnostic cutoff values for occludable angle detection. This is useful for angle closure glaucoma diagnosis and management. Moreover, 840 nm FD-OCT is a useful tool for postsurgical evaluation in glaucoma patients.

## 1. Introduction

Since its initial development approximately two decades ago [1], optical coherence tomography (OCT) has become an indispensable tool in ophthalmic imaging. Although it was initially designed to image the posterior segment and retina [2, 3], OCT's capability to generate cross-sectional images of internal biological structures without contacting the ocular surface was quickly adapted to visualize the anterior segment and cornea [4–7]. Anterior segment OCT technology has been improved a great deal since Izatt et al. [4] first reported using OCT to image the anterior segment of the eye in 1994. Of the many uses that have come out of those improvements, one of the most commonly cited is the evaluation of the anterior chamber angle for narrow-angle glaucoma [8]. 

## 2. Anterior Segment OCT 

**Figure 1:**
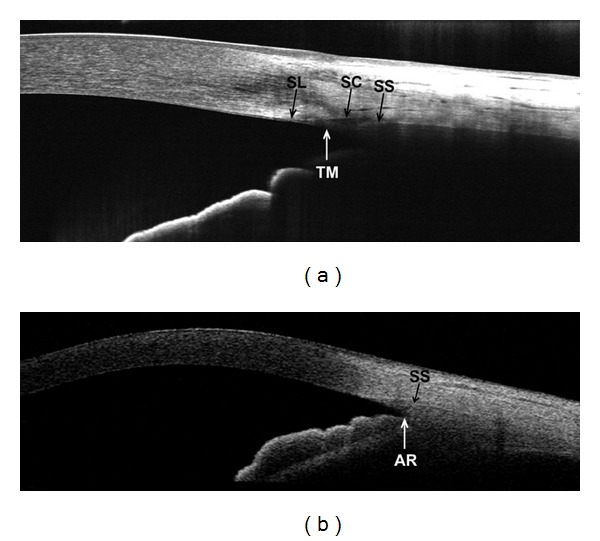
Two scans of the same nasal angle in a normal subject. (a) Fourier-domain RTVue OCT image using a 6 mm CL-Angle scan pattern. Schwalbe's line (SL), trabecular meshwork (TM), Schlemm's canal (SC), and scleral spur (SS) are visible. However, the iris root (IR) and angle recess (AR) are not visible in this image. (b) Time-domain Visante OCT image using a 10 mm high-resolution corneal scan pattern. In this image, both the scleral spur and angle recess are visible, but the trabecular meshwork, Schlemm's canal, and Schwalbe's line are not resolvable.

**Figure 2:**
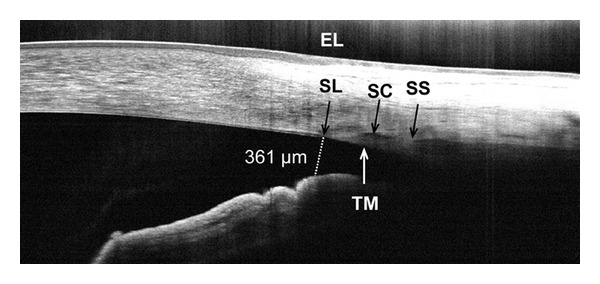
Frame-averaged cross-sectional OCT image of the nasal angle in a normal subject. The high resolution helps to visualize the termination of the endothelium and Descemet's membrane (Schwalbe's line, SL), which is a useful landmark on these images. Also visible are the external limbus (EL), Schlemm's canal (SC), and the trabecular meshwork (TM). The scleral spur (SS) is faintly visible in this case. The angle recess, iris root, and ciliary body are not visible due to blocking by the sclera. The angle opening distance between SL and the anterior surface of the iris (AOD-SL, dotted line) was 361 *μ*m, indicating that the angle is open.

**Figure 3:**
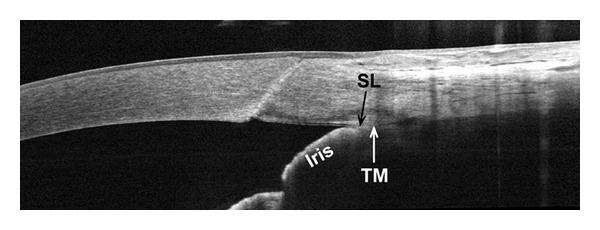
Frame-averaged cross-sectional OCT image of a closed angle with modified Shaffer grade of 0 by gonioscopy. The high definition of the image allows the visualization of the Schwalbe's line (SL) and the contact between the iris and the trabecular meshwork (TM). The AOD-SL is obviously zero in this case.

**Figure 4:**
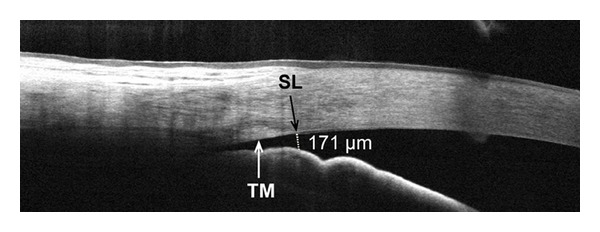
Frame-averaged cross-sectional OCT image of the nasal angle in an eye with primary narrow angle glaucoma. The AOD-SL (dotted line) was measured at 177 *µ*m, below the diagnostic cutoff value of 230–290 *μ*m, indicating a potentially occluded angle. This agreed with a gonioscopic grade of 1 on the modified Shaffer scale. The trabecular meshwork (TM) and Schwalbe's line (SL) can be distinguished.

**Figure 5:**
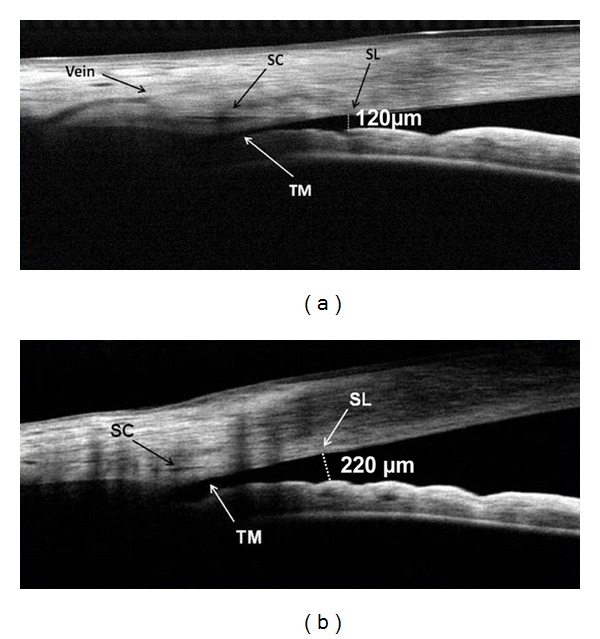
Pre- and postlaser peripheral iridotomy OCT images of an eye with narrow angles. (a) Preoperative image of the nasal angle with frame averaging. The short distance between the trabecular meshwork (TM) and the iris indicates a narrow, potentially occluded angle. The AOD-SL (dotted line) measured 120 *μ*m. An aqueous collector vein and the Schlemm's canal (SC) are also visible. (b) Frame-averaged cross-sectional OCT image of the same angle following laser peripheral iridotomy. The procedure nearly doubled the AOD-SL (dotted line) to 220 *μ*m, reprinted with permission from SLACK Incorporated: Huang D, Duker JS, Fujimoto JG, Lumbroso B, Schuman JS, Weinreb RN. *Imaging the Eye from Front to Back with RTVue Fourier-Domain Optical Coherence Tomography.* Thorofare, NJ: SLACK Incorporated; 2010.

**Figure 6:**
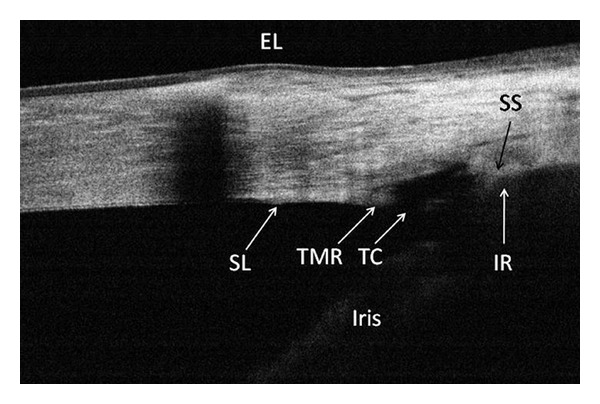
A cross-sectional OCT image of the nasal angle following trabectome surgery. This frame-averaged image shows that the posterior trabecular meshwork has been removed, leaving a 374 *μ*m wide trabecular cleft (TC) and an anterior trabecular meshwork remnant (TMR). Although scleral shadowing has caused the iris root (IR) to appear indistinct, it was possible to trace its position by contiguity with the iris. The limbal girdle of Vogt is the cause of the shadowing in the peripheral cornea. (Courtesy of Brian A. Francis, MD, USA), reprinted with permission from SLACK Incorporated: Huang D, Duker JS, Fujimoto JG, Lumbroso B, Schuman JS, Weinreb RN. *Imaging the Eye from Front to Back with RTVue Fourier-Domain Optical Coherence Tomography.* Thorofare, NJ: SLACK Incorporated; 2010.

There are currently two types of OCT instruments used for imaging the anterior segment: time domain (TD) and the more recently developed Fourier domain (FD). Relying on the mechanical movement of the reference mirror to produce each axial scan (A-scan), TD-OCT samples the range of depth being imaged one point at a time. This serial sampling limits the speed of image acquisition. In contrast, FD-OCT—also known as spectral-domain (SD) OCT, spectral OCT, high-definition (HD) OCT, and frequency-domain OCT—does not rely on the mechanical movement of a reference mirror: the reflections from the entire depth range being imaged are sampled simultaneously. The interference between the sample and reference beams is detected as a spectral interferogram, which undergoes Fourier transform to produce axial scans (A-scans) [9]. The parallel detection greatly improves speed without sacrificing signal level. Therefore, FD-OCT instruments can provide scan speeds 10–100 times faster than TD-OCT instruments [10]. The faster speeds minimize the effect of eye movements during imaging and allow higher-definition imaging due to denser axial scans in the same transverse scan length. The scanning speed of FD-OCT also facilitates the registration and averaging of sequential frames. This frame averaging makes it possible to both increase the signal-to-noise ratio of an image and sharpen the anatomic features contained therein (Figures 1, 2, 3, 4, 5, and 6). 

Anterior segment OCT systems can also be differentiated by the central wavelengths they utilize for scans. Current OCT instruments commonly use 840 nm, 1050 nm, or 1310 nm wavelength light. The use of longer wavelength light to image the anterior chamber angle does have advantages. 1310 nm and 1050 nm lights show lower scattering and signal loss in turbid media [11, 12], permitting much better penetration through the limbus and sclera than is possible using shorter wavelengths. 

This increased penetration allows 1310 nm wavelength OCT to accurately measure gross angle morphology and visualize angle structures such as the iris root and scleral spur [13–15]. As a result, past studies used 1310 nm OCT systems and developed quantitative angle parameters using scleral spur as the anatomical landmark [16–19]. The axial resolutions of the earlier OCT systems used in the abovementioned studies were limited to 15–20 *μ*m and did not allow reliable identification of smaller angle structures such as the trabecular meshwork and Schwalbe's line. Newer OCT systems capable of producing axial resolutions of 1–5 *μ*m are now available [20, 21]. This marked increase in resolution is due to the combination of broader bandwidth and shorter wavelength. 

The 840 nm FD-OCT systems discussed in this paper had an axial resolution of 5 *μ*m and were able to visualize details of the anterior segment that could not be resolved with previous OCT systems [14, 16]. These details include the Schwalbe's line, Schlemm's canal, trabecular meshwork, and aqueous collector veins (Figures 1–6). As an example, we used both the 840 nm RTVue FD-OCT (Optovue, Inc. Fremont, CA, USA) and the 1310 nm Visante TD-OCT (Carl Zeiss Meditec, Inc.; Dublin, CA) to image the same anterior chamber angle location of a normal volunteer. We then compared the resulting images (Figure 1). The 1310 nm Visante OCT had better penetration than the RTVue and could visualize both scleral spur and angle recess. The device's resolution, however, prevented visualization of fine anatomical structures such as Schwalbe's line, Schlemm's canal, and trabecular meshwork. By contrast, the higher resolution 840 nm RTVue OCT image showed Schwalbe's line, trabecular meshwork, Schlemm's canal, and scleral spur (Figure 1), but provided limited visualization of the angle recess. In summary, the Visante provided a wider field of view and better penetration, but with lower resolution, while the RTVue provided higher-resolution information on a narrower portion of the anterior chamber. Wylegała et al. [22] reported similar observations in a 2009 study comparing the results of anterior segment imaging with the RTVue and the Visante. 

The study examined 54 eyes and compared measurements of central corneal thickness (CCT), trabecular-iris area (TISA), angle opening distance at the scleral spur (AOD-SS), and anterior segment morphology. Upon comparing the mean values of the CCT, TISA, and AOD-SS measurements obtained using the RTVue with those obtained using the Visante, Wylegała et al. found no statistically significant difference between the measurements. There was, however, a difference in the amount of anatomic detail contained in the images. The study found that the RTVue images showed structures, such as Bowman's layer and Schlemm's canal, which could not be visualized with the Visante.

## 3. Schwalbe's Line-Based Quantitative Angle Assessment with High-Resolution FD-OCT

Schwalbe's line represents the termination of Descemet's membrane and is approximately 500–750 *μ*m anterior to the scleral spur. The distance between Schwalbe's line and the scleral spur represents the trabecular meshwork and the aqueous humor filtration distance. Schwalbe's line was proposed as an anatomical landmark because it could be consistently identified in 840 nm FD-OCT images. Several research groups recommended measuring angle opening distance at the Schwalbe's line (AOD-SL) for quantitative angle assessment [23–25]. Further, AOD-SL has demonstrated good repeatability and strong correlation with gonioscopic grading. 

Cheung et al. [24] recruited 73 participants from glaucoma clinics at the Singapore National Eye Center. Each participant underwent dark-room gonioscopy, and the angle was graded in four quadrants (superior, inferior, nasal, and temporal) using the modified Shaffer grading system. Each quadrant was defined as occludable if the posterior trabecular meshwork was not visible. OCT imaging was performed on the nasal and temporal quadrants of each eye using the Cirrus HD-OCT model 4000 (software version 3.0, Carl Zeiss Meditec, Dublin, CA, USA). An external fixation light was used to guide each patient's fixation to the side of the OCT instrument during imaging. The illumination conditions of the room during scanning were not reported. One eye from each subject was selected randomly for statistical analysis. Schwalbe's line was identified in 95% of angle scans. The AOD-SL measurements showed consistent interobserver and intraobserver reliability with intraclass correlation coefficients of 0.979 and 0.988, respectively. There was also strong correlation between AOD-SL measurements and gonioscopic grading, with a Spearman correlation coefficient of 0.709. The occludable angle AOD-SL measurements ranged from 94 *μ*m to 172 *μ*m, and the nonoccludable angle AOD-SL measurements ranged from 286 *μ*m to 347 *μ*m. Although Cheung et al.did not report a diagnostic cutoff for occludable angle, based on these results one could be estimated at approximately 230 *μ*m. 

In a similar study, Qin et al. [25] recruited 35 glaucoma patients from the Doheny Eye Institute at the University of Southern California, Los Angeles, California, USA. Each subject underwent dark-room gonioscopic examination and a modified Shaffer grade was recorded for each of four quadrants. An occludable angle was defined as Grade 1 or lower in all quadrants. Angle scans were performed under standardized dim illumination conditions (6.6 foot candles) using the RTVue FD-OCT (Optovue, Inc., Fremont, CA, USA). Schwalbe's line was visualized in 97.7% of eyes scanned. Qin et al. also reported good repeatability for AOD-SL measurements with intraobserver coefficients of variation ranging from 9.8% to 12.0% and interobserver coefficients of variation ranging from 10.1% to 13.3%. Spearman's rho analysis showed strong correlation between AOD-SL measurements and gonioscopic grading with correlation coefficients of 0.80 for nasal angles and 0.81 for temporal angles. The occludable angle AOD-SL measurements ranged from 0 *μ*m to 350 *μ*m, and the nonoccludable angle measurements ranged from 192 *μ*m to 922 *μ*m. Qin et al. determined a diagnostic cutoff value for occludable angle of 290 *μ*m by performing receiver operating characteristic (ROC) analyses. 

## 4. Postoperative Angle Imaging with FD-OCT

Surgical intervention is an important aspect of glaucoma management. Pre- and post-operative angle imaging with FD-OCT is useful for documenting and evaluating surgery outcomes. For example, using FD-OCT to image the angle both before and after laser peripheral iridotomy made it possible to document the resulting angle morphological changes (Figure 5) [26]. This technology was also used to evaluate filtering blebs and demonstrate subconjunctival filtration after trabeculotomy [27], and to confirm the excision of trabecular tissue after trabectome treatment (Figure 6) [26]. OCT is also used to examine iridotrabecular contact as a peripheral anterior synechia after penetrating keratoplasty [28].

## 5. Conclusion

High-resolution 840 nm FD-OCT is capable of providing angle images with fine anatomical structures. Studies showed that the Schwalbe's line could be identified in over 95% of FD-OCT angle scans. In addition, measurements of the angle opening distance at the Schwalbe's line were shown to have good repeatability and strong correlation with gonioscopic assessments. Quantitative angle measurements using Schwalbe's line as the anatomical landmark may help clinicians to better assess and manage narrow-angle glaucoma. Based on gonioscopic correlation, studies so far indicate the AOD-SL diagnostic cutoff for occludable angle is between 230 *μ*m and 290 *μ*m. Therefore, measurement of angle opening distance with FD-OCT may be useful in the assessment of angle closure risk. After filtration surgery, FD-OCT is also a useful tool for postsurgical evaluation of angle structures.
